# Transcriptome of melanoma cells from two mouse models, *Tyr:NRas*^*Q61K*^and *Tyr:Rack1-HA, Tyr:NRas*^*Q61K*^

**DOI:** 10.1016/j.dib.2017.07.006

**Published:** 2017-07-11

**Authors:** Cécil Campagne, Stéphanie Pons, Diane Esquerre, Jordi Estellé, Emmanuelle Bourneuf, Uwe Maskos, Giorgia Egidy

**Affiliations:** a INRA, UMR955 Génétique Fonctionnelle et Médicale, Ecole Nationale Vétérinaire d’Alfort, F-94704 Maisons-Alfort, France; b Université Paris-Est, Ecole Nationale Vétérinaire d’Alfort, UMR955 Génétique Fonctionnelle et Médicale, F-94704 Maisons-Alfort, France; c Unité Neurobiologie Intégrative des Systèmes Cholinergiques, URA2182, CNRS, Institut Pasteur, F75724 Paris Cedex 15, France; d GenPhySE, Université de Toulouse, INRA, INPT, ENVT, Castanet Tolosan, France; e GABI, INRA, AgroParisTech, Université Paris-Saclay, F-78352 Jouy-en-Josas, France; f LREG, CEA, Université Paris-Saclay, F-78352 Jouy-en-Josas, France

**Keywords:** Cutaneous melanoma, RACK1, shRNA, RNAseq

## Abstract

The transcriptome sequencing of melanoma cells from two mouse models differing in the expression level of the scaffold protein Receptor for activated C kinase (RACK1) are presented. Primary melanoma cells were harvested from *Tyr:NRas*^*Q61K*^*; Pax3*^*GFP/+*^ mice, with or without the *Tyr:Rack1-HA* transgene. Cells were cultured and infected with scramble shRNA or *Rack1*-targeting shRNA*,* on technical triplicates of viral infection. Libraries were prepared by selecting polyadenylated mRNAs and RNA Sequencing (RNASeq) was performed. Samples are described in the SRA portal (SRP096162) and FASTQ files have been deposited in Sequence Read Archive (accession numbers: SRR5150106 to SRR5150117). The interpretation of these data is presented in the following research article: “RACK1 cooperates with NRAS^Q61K^ to promote melanoma *in vivo*” (Campagne et al., 2017, doi: 10.1016/j.cellsig.2017.03.015) [1].

## **Specifications Table**

**Table:** 

Subject area	Biology
More specific subject area	Oncology
Type of data	Sequence data
How data was acquired	High throughput RNA sequencing
Data format	FASTQ files
Experimental factors	Cells were infected by Scramble shRNA or shRNA targeting *Rack1*
Experimental features	Cells were cultured as previously described [2], then infected with lentiviral vectors containing scramble or *Rack1*-targeting shRNA for three days before RNA collection.
Data source location	Maisons-Alfort, France
Data accessibility	Sample description has been deposited in the BioSample Submission Portal as Bioproject PRJNA360045 and the full data sets have been submitted to NCBI Sequence Read Archive (SRA, https://www.ncbi.nlm.nih.gov/sra) under accession numbers SRR5150106 to SRR5150117. Data are publicly available.

## **Value of the data**

• These data reflect the whole transcriptome of melanoma cells from two mouse models and can be used to explore the basal RNA expression of transformed melanocytes.
• The data can be used to understand *Rack1* modulation effect *in vivo.*
• The data can be used to gain insight into molecular mechanisms modifying latency and incidence of melanoma in mice.

## 1 Data

Data provided in this article correspond to the FASTQ files obtained after RNA sequencing of melanoma cells from two mouse models, treated with scramble shRNA or *Rack1*-targeting shRNA.

## 2 Experimental design, materials and methods

### 2.1 Cell culture and RNA interference

Melanoma cells were sampled from primary tumors of *Tyr:NRas*^*Q61K*^*; Pax3*^*GFP/+*^ mice with or without *Tyr:Rack1-HA* transgene. Cells were cultured as previously described [2], and RNA interference was performed with scramble shRNA (sequence GTCACTCACCCTTCGGTTATT) or a shRNA targeting exon 2 of *Rack1* (sequence GGTCACTCCCACTTCGTTATT). Transduction was performed at 0.45 ng/µl of lentiviral titer in the presence of polybrene. RNAs were collected on the third day.

### 2.2 RNA sequencing and data analysis

RNA samples were sequenced in an Illumina HiSeq. 2500 following manufacture׳s recommendations. In brief, libraries were prepared by selecting polyadenylated mRNA using the TruSeq RNA Sample Prep Kit (Illumina, San Diego, CA). Samples were tagged with barcode sequences for subsequent identification, amplified by PCR and quantified by qPCR using the QPCR Library Quantification Kit (Agilent Technologies, Santa Clara, CA). 4 libraries were sequenced in paired-ends 2×100 bp in three Illumina lanes. The full data sets have been submitted to NCBI Sequence Read Archive (SRA) under Accession.
